# Analysis of Germline GLI1 Variation Implicates Hedgehog Signalling in the Regulation of Intestinal Inflammatory Pathways

**DOI:** 10.1371/journal.pmed.0050239

**Published:** 2008-12-09

**Authors:** Charlie W Lees, William J Zacharias, Mark Tremelling, Colin L Noble, Elaine R Nimmo, Albert Tenesa, Jennine Cornelius, Leif Torkvist, John Kao, Susan Farrington, Hazel E Drummond, Gwo-Tzer Ho, Ian D. R Arnott, Henry D Appelman, Lauri Diehl, Harry Campbell, Malcolm G Dunlop, Miles Parkes, Sarah E. M Howie, Deborah L Gumucio, Jack Satsangi

**Affiliations:** 1 Gastrointestinal Unit, Molecular Medicine Centre, University of Edinburgh, Edinburgh, United Kingdom; 2 Immunobiology Group, Medical Research Council (MRC) Centre for Inflammation Research, Queens Medical Research Institute, University of Edinburgh, Edinburgh, United Kingdom; 3 Department of Cell and Developmental Biology, University of Michigan Medical School, Ann Arbor, Michigan, United States of America; 4 Inflammatory Bowel Disease (IBD) Research Group, Addenbrooke's Hospital, University of Cambridge, Cambridge, United Kingdom; 5 Colon Cancer Genetics Group, MRC Human Genetics Unit, Western General Hospital, Edinburgh, United Kingdom; 6 Department of Pathology, Genentech, Inc, South San Francisco, California, United States of America; 7 Department of Medical & Surgical Gastroenterology, Karolinska University Hospital, Stockholm, Sweden; 8 Department of Internal Medicine, University of Michigan Medical School, Ann Arbor, Michigan, United States of America; 9 Department of Pathology, University of Michigan Medical School, Ann Arbor, Michigan, United States of America; University of Oslo, Norway

## Abstract

**Background:**

Ulcerative colitis (UC) and Crohn's disease (CD) are polygenic chronic inflammatory bowel diseases (IBD) of high prevalence that are associated with considerable morbidity. The hedgehog (HH) signalling pathway, which includes the transcription factor glioma-associated oncogene homolog 1 (GLI1), plays vital roles in gastrointestinal tract development, homeostasis, and malignancy. We identified a germline variation in *GLI1* (within the IBD2 linkage region, 12q13) in patients with IBD. Since this IBD-associated variant encodes a GLI1 protein with reduced function and our expression studies demonstrated down-regulation of the HH response in IBD, we tested whether mice with reduced Gli1 activity demonstrate increased susceptibility to chemically induced colitis.

**Methods and Findings:**

Using a gene-wide haplotype-tagging approach, germline *GLI1* variation was examined in three independent populations of IBD patients and healthy controls from Northern Europe (Scotland, England, and Sweden) totalling over 5,000 individuals. On log-likelihood analysis, *GLI1* was associated with IBD, predominantly UC, in Scotland and England (*p* < 0.0001). A nonsynonymous SNP (rs2228226C→G), in exon 12 of *GLI1* (Q1100E) was strongly implicated, with pooled odds ratio of 1.194 (confidence interval = 1.09–1.31, *p* = 0.0002). *GLI1* variants were tested in vitro for transcriptional activity in luciferase assays. Q1100E falls within a conserved motif near the C terminus of GLI1; the variant GLI protein exhibited reduced transactivation function in vitro. In complementary expression studies, we noted the colonic HH response, including GLI1, patched (PTCH), and hedgehog-interacting protein (HHIP), to be down-regulated in patients with UC. Finally, *Gli1^+/lacZ^* mice were tested for susceptibility to dextran sodium sulphate (DSS)-induced colitis. Clinical response, histology, and expression of inflammatory cytokines and chemokines were recorded. *Gli1^+/lacZ^* mice rapidly developed severe intestinal inflammation, with considerable morbidity and mortality compared with wild type. Local myeloid cells were shown to be direct targets of HH signals and cytokine expression studies revealed robust up-regulation of IL-12, IL-17, and IL-23 in this model.

**Conclusions:**

HH signalling through GLI1 is required for appropriate modulation of the intestinal response to acute inflammatory challenge. Reduced GLI1 function predisposes to a heightened myeloid response to inflammatory stimuli, potentially leading to IBD.

## Abstract 2

Charlie Lees and colleagues identify a reduced-function variant of the hedgehog signaling pathway protein GLI1 that associates with inflammatory bowel disease, and investigate its role in a mouse model of colitis.

## Editors' Summary

**Background.:**

Inflammatory bowel diseases (IBDs) are common disorders in which parts of the digestive tract become repeatedly or continuously inflamed. The immune system normally protects the body from entities it identifies as foreign, but in IBD it mistakenly recognizes gut tissue, and immune system cells accumulate in the lining of the bowel, which causes inflammation. There are two main types of IBD—Crohn's disease (CD), which mainly affects the small bowel, and ulcerative colitis (UC), which affects only the large bowel (colon). Both types tend to run in families and usually develop between the ages of 15 and 35 years. Symptoms—including diarrhea, abdominal cramps, and unexplained weight loss—can be mild or severe and the disease can develop slowly or suddenly. There is no cure for IBD except surgical removal of the affected part of the digestive tract. However, drugs that modulate the immune system (for example, corticosteroids) or that specifically inhibit “proinflammatory cytokines” (proteins made by the immune system that stimulate inflammation) are often helpful in reducing symptoms.

**Why Was This Study Done?:**

Why the immune system becomes unbalanced in people with IBD is not clear but it is known that IBD is “polygenic,” that is, a disease caused by the combined actions of two or more inherited gene variants. Although UC and CD are clinically different diseases, they share several “susceptibility loci” (regions of the genome that harbor disease-associated gene variants), including the IBD2 locus. The identification of the actual gene within the IBD2 locus that is altered in people who are susceptible to IBD might provide new insights into what causes the immune imbalance in IBD and into how to treat the disease. In this study, the researchers test the hypothesis that a variant of a gene called *GLI1*, which lies in the IBD2 locus, is associated with IBD susceptibility. *GLI1* encodes a transcription factor (a protein that regulates the production of proteins) that is a central component in the signaling pathway named for a protein called “hedgehog.” This pathway is involved in the development of many organs, including the digestive tract.

**What Did the Researchers Do and Find?:**

The researchers used a technique called gene-wide haplotype tagging to look for inherited *GLl1* variants in patients with IBD and in healthy people living in Scotland, England, and Sweden. A specific variant of the *GLI1* gene, resulting in alteration of a single amino acid component of the GLI1 protein, was associated with IBD (particularly with UC) in both Scotland and England; the same variant was weakly associated with IBD in the Swedish population. The variant GLI1 protein was only half as active as the normal protein in a laboratory assay, and, consistent with this result, the expression of several components of the hedgehog signaling pathway was lower in colon samples taken from patients with UC than in samples taken from healthy individuals. Finally, *Gli1^+/lacZ^* mice (which express half the normal amount of Gli1 protein) developed severe intestinal inflammation more rapidly than wild-type mice when they were treated with dextran sodium sulfate (DSS), a chemical that induces acute (sudden) colitis. Cellular analysis revealed that myeloid cells (cells that sense and modify the inflammatory response) are direct targets of the hedgehog signaling pathway. Furthermore, the expression of several pro-inflammatory cytokines (in particular, one called IL-23) increased more markedly in the *Gli1^+/lacZ^* mice than in the wild-type mice after DSS treatment.

**What Do These Findings Mean?:**

These findings suggest that the normal response of the mammalian gut to challenge with inflammatory substances involves hedgehog signaling through GLI1 and that reduced GLI1 function might be one trigger for IBD. More specifically, the human genetic studies identify a *GLI1* variant that is associated with IBD (at least in certain north European populations), the laboratory experiments indicate that this *GLI1* variant encodes a protein with reduced activity, and the animal studies show that a similar reduction in Gli1 activity is sufficient to heighten intestinal inflammatory responses. Although this last result needs to be confirmed in animal models of chronic colitis that more closely resemble human IBD, these findings suggest that drugs that modulate hedgehog signaling might be useful in the treatment of IBD.

**Additional Information.:**

Please access these Web sites via the online version of this summary at http://dx.doi.org/10.1371/journal.pmed.0050239.

- The MedlinePlus Encyclopedia has pages on Crohn's disease and on ulcerative colitis (in English and Spanish)
- MedlinePlus provides links to other information Crohn's disease and ulcerative colitis (in English and Spanish)
- The US National Institute of Diabetes and Digestive and Kidney Diseases provides information on Crohn's disease and ulcerative colitis
- The UK National Health Service Direct Encyclopedia also provides information on Crohn's disease and on ulcerative colitis
- Wikipedia has a page on the hedgehog signaling pathway (note: Wikipedia is a free online encyclopedia that anyone can edit; available in several languages)

## Introduction

Ulcerative colitis (UC; Mendelian Inheritance in Man [MIM] 191390) and Crohn's disease (CD; MIM 266600) are chronic, relapsing, inflammatory bowel diseases (IBD) of high prevalence (200–400 cases per 100,000 in Northern Europe and North America [1]) and are associated with considerable morbidity. Precise aetio-pathogenetic mechanisms are not understood but several lines of evidence implicate the central importance of a dysregulated host response to intestinal bacteria [2]. Epidemiological data, detailed molecular studies, and recent genome-wide association studies strongly suggest that UC and CD are related polygenic diseases that share some susceptibility loci (*IL-23R*, *IL-12B*, and *NKX2.3*), but differ at others: *NOD2*, *ATG16L1*, and *IRGM* are specific CD genes; the *ECM1* locus is associated with UC [3–10]. The IBD2 locus (Online Mendelian Inheritance in Man [OMIM] 601458**)** on Chromosome 12q13 was first identified in a UK genome-wide linkage scan (peak LOD score 5.47 at D12S83) [11] involving both UC and CD patients. Later studies showed that IBD2 contributes significantly to UC, notably extensive disease, but perhaps in a lesser way to CD susceptibility [12,13].

A strong candidate gene that maps to the IBD2 locus is *GLI1*, one of three related GLI transcription factors that transduce secreted hedgehog (HH) signals. HH signalling is key in gut development, homeostasis, and malignancy, but has not been carefully studied in IBD [14]. In developing intestine, Sonic (SHH) and Indian Hedgehog (IHH) provide a paracrine signal from epithelium to the mesenchymally expressed receptor patched (PTCH) [14]. The binding of HH to PTCH releases the membrane protein smoothened (SMO) from inhibition and allows HH signal transduction through the zinc finger transcription factors glioma-associated oncogene homolog 1 (GLI1), GLI2, and GLI3 to direct tissue patterning and cell fate (Figure S1) (see [14] and citations therein). Chronic injury, inflammation, and repair are critical aspects of IBD, and thus it is pertinent that the HH pathway is centrally involved in these processes in several other tissues, including muscle [15], liver [16,17], and lung [18,19]. Indeed, HH signalling may play a central role in the inflammatory response since SHH is critical for T lymphocyte development [20], adult human CD4+ T cell activation [21,22], and myeloid cell maturation in the spleen [23]. Furthermore, SHH has recently been suggested to be a direct transcriptional target of NF-κB [24]. Dysregulation of components of the HH pathway has also been noted in inflammatory diseases of the gut, including Barrett oesophagus, chronic gastritis, and IBD [25]. Using microarray gene expression analyses of colonoscopic biopsies, we recently demonstrated that GLI1 expression is greater in the distal compared with the proximal colon, and is down-regulated in the intestinal mucosa in inflamed UC compared with noninflamed samples [26].

Taken together, these studies suggested a possible association between GLI1 and IBD susceptibility, and we set out to directly test this possibility in both human disease and murine model systems. Our main initial objectives were to ascertain, from human genetic association study data, whether germline *GLI1* variation was associated with IBD, and to describe the expression of HH signalling components in colonic inflammation in humans. These results led us to hypothesize that a reduced dosage of functional GLI1 protein might play an important role in colonic inflammation. To test this hypothesis directly, we challenged *Gli1^+/lacZ^* mice and their wild-type (WT) littermates with dextran sodium sulphate (DSS) to induce acute intestinal inflammation, and monitored clinical and histological parameters, cytokine profiles, and cellular targets of HH signalling in vivo.

## Methods

Written, informed consent was obtained from all patients and controls. The study protocol was approved by the Lothian Research and Ethics Committee (LREC-2000/4/192 and 2004/S1103/22); Cambridge (LREC-01/418; MREC-03/5/012); and Regional Ethics Committee, Karolinska Institutet***.***

### Participants and Samples

#### Individuals for genotyping.

In Scotland the population consisted of 817 IBD cases (474 UC; 335 CD; eight colonic IBD type unclassified [IBDU]) and 1,374 healthy controls (HC) (Table 1). Diagnosis was made by clinical, radiological, and histopathological means, adhering to the criteria of Lennard-Jones [27]. All IBD patients attended the clinic at the Western General Hospital (Edinburgh, Scotland), a tertiary referral centre for IBD in southeast Scotland. The population was 98.5% white, non-Jewish. The median age at diagnosis was 31.0 y (IQR 23.3–46.0). Controls were recruited from across Scotland; median age was 50.0 (IQR 43.0–55.0), ethnicity >99% white, non-Jewish, and 48.7% male sex.

**Table 1:**
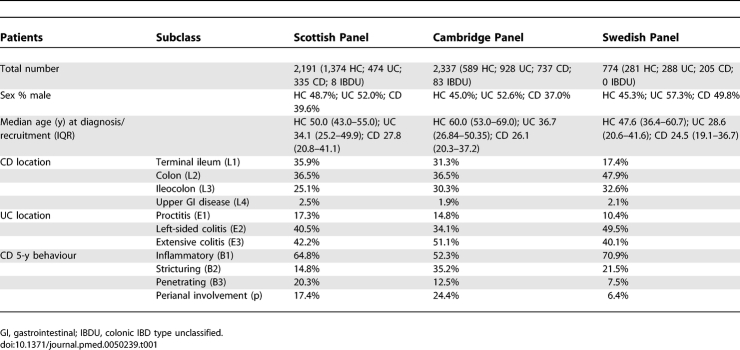
Detailed Demographics and Phenotypic Data on Scottish, Cambridge, and Swedish IBD Population

In Cambridge, England 1,748 unrelated Caucasian IBD patients (Lennard-Jones criteria) of northern European origin attending IBD clinics in East Anglia, UK were recruited comprising 928 UC, 737 CD, and 83 with IBDU. The population was >99% white, non-Jewish. The 589 ethnically and geographically matched HCs were previously recruited in East Anglia for the European Prospective Investigation of Nutrition and Cancer (EPIC). Median age of controls was 60 y.

In Sweden 288 UC and 205 CD patients (Lennard-Jones criteria) were recruited from various hospitals in Stockholm County. 281 Swedish HC were healthy volunteers from the Karolinska University Hospital staff (*n* = 170) and orthopaedic day-case surgery patients (*n* = 111) with no previous medical conditions. Cases and controls were >99% white, non-Jewish.

#### Phenotyping*.*

Phenotypic data were collected by patient questionnaire, interview, and case note review, and classified according to Montreal criteria (Table 1) [28].

### Genotyping

#### Scotland and Sweden.

Genomic DNA was extracted from blood samples using a modified salting-out technique as previously described, and Nucleon kits. Genotypes were derived using the Taqman system for allelic discrimination; the assays were available from Applied Biosystems as Taqman SNP Genotyping Assays (7900HT sequence detection system; Applied Biosystems), except for SNPs rs10783819, rs3809114, rs507562, rs542278, rs730560, rs1669296, and rs775322, which were genotyped on the Illumina platform. The accuracy of each Taqman assay was checked by repeat analysis in 5% of cases, with 100% concordance. Genotype distributions in all populations were consistent with Hardy-Weinberg equilibrium (HWE) (*p* > 0.01) for all SNPs. For the four tagging SNPs (tSNPs) that could not be derived by Taqman, genotypes were obtained by direct sequencing.

#### Cambridge.

DNA was extracted using Nucleon kits. Genotyping of CD cases and controls was performed using the Taqman biallelic discrimination system using an ABI 7900HT analyser (Applied Biosystems). Genotyping of UC cases was performed using a 1,536-SNP Golden Gate bead array (Illumina). Concordance between platforms was assessed by genotyping 92 UC cases for SNPs rs2228224 and rs2228226 with concordance rates of 100% and 97.9% respectively and no evidence of systematic bias between platforms. Genotype distributions in case and control populations were consistent with HWE (*p* > 0.01) for all SNPs.

### Gene Expression by Microarray and Quantitative Reverse-Transcription PCR

The cohort of patients used in the microarray studies consisted of 67 patients with UC, 53 with CD, and 31 HCs. For UC patients, eight were recruited at diagnosis (treatment naïve), 18 had active disease, and 41 quiescent disease; ten were on systemic corticosteroid therapy, 11 on immunosuppressants (azathioprine, mercaptopurine, or methotrexate), and 40 on 5-amino salicylic acid (5-ASA) therapy. Four of 53 patients with CD were on corticosteroids, 13 of 53 on immunosuppressants, and 21 of 53 on a 5-ASA. Eight of 31 HC had non-IBD inflammation: two scattered lymphoid aggregates with history of gastroenteritis, two microscopic colitis, one pseudomembranous colitis, one diverticulitis, one amoebiasis, and one eosinophilic infiltrate.

The assessment of RNA quality and integrity was performed for the microarray experiments as follows: the amplified cRNA was purified using the RNeasy Mini Kit protocol (Qiagen) and 1 μl of amplified cRNA was quantified using the NanoDrop ND-1000 Spectrophotometer. The distribution of log intensities for each sample was plotted and outlier samples (i.e., greater than two standard deviations from the mean) were excluded from analysis*.* For additional demographics, phenotyping, and full methodology pertaining to the generation of the microarray dataset and quantitative reverse-transcription PCR (QPCR) see Noble et al [26].

### Induction of DSS-Induced Colitis and Histological Analysis

Groups of two to four WT *Gli1^+/lacZ^* or WT littermate controls in mixed cages (bred at least three generations onto a C57BL/6 background) were administered 3% DSS in drinking water for 4–6 d. The amount of DSS consumed was not significantly different between WT and *Gli1^+/lacZ^* animals (unpublished data). *Gli1^+/lacZ^* animals tended to weigh more than their WT littermates (mean, 28 g for *Gli1^+/lacZ^* animals and 24 g for WT animals). Therefore, DSS-treated weight matched WT C57Bl/6 animals (*n* = 4) were also tested along with *Gli1^+/lacZ^* animals and WT littermates. No differences were evident between WT littermates and weight matched C57Bl/6. All animals were monitored daily for diarrhoea, bloody stool, and weight loss. Clinical scoring was as follows: 0, no symptoms; 1, diarrhoea; 2, bloody stool; 4, severe rectal bleeding and morbidity to the point of immobility/death. For histology, a segment of large intestine tissue of equal length and location for all animals was fixed overnight in 4% paraformaldehyde, dehydrated, infiltrated with paraffin, and sectioned at 5 μm. The remaining colonic tissue was taken for RNA analysis or frozen sectioning and Gli1-LacZ expression analysis. Slides were stained with haematoxylin/eosin and scored histologically by a gastrointestinal pathologist (H.D.A.) blinded to the source of the tissue. Colitis scores were calculated using published methods [29].

### Gli1-LacZ Immunohistochemistry

Colonic tissue was dissected out in cold PBS and fixed for 30 min in 4% PFA at 4 °C. After a brief rinsing in PBS, the tissue was incubated in 30% sucrose/PBS at 4 °C overnight and then embedded in OCT and frozen on dry ice.

Immunohistochemistry was performed on 8- to 10-μm frozen sections. Sections were dried, rinsed in PBS, and blocked for 1 h in 10% normal goat serum, 0.1% BSA, and 0.3% Triton-X in TBS (50 mM Tris-HCl [pH 7.4], 150 mM NaCl). Antibodies used were: hamster anti-CD3 (Serotec, 1:500), rat anti-CD19 (Serotec, 1:500), goat anti-CD11b (Abcam 1:500), FITC-conjugated anti-CD11c (Abcam 1:250), and rabbit anti–β-galactosidase (a gift of J. Douglas Engel, Department of Cell and Developmental Biology, University of Michigan, 1:2,000). All antibody staining was performed over night at 4 °C, followed by incubation with fluorophore-labeled Alexafluor secondary antibodies from Molecular Probes, 1:1,000 for 1 h. Nuclei were counterstained with DAPI, and slides were mounted in Prolong Gold Antifade Reagant (Invitrogen). Images were taken using an Olympus BX51 at 200× for immunofluorescence and an Olympus FV500 confocal microscope at 1,000× for 0.30-μm optical sectioning.

### GLI1 Protein Mutagenesis, Luciferase Assay, and Immunofluorescence

GLI1 E1100 was amplified from Image Clone number 3531657, and cloned into pCMV-Tag2b, adding an N-terminal FLAG motif. GLI1 Q1100 was obtained by inducing a point mutation using the QuikChange II Site-Directed Mutagenesis Kit (Stratagene) following the manufacturer's protocol. Plasmids encoding 8xGli-Luciferase, m8x Gli-Luciferase (mutated Gli sites), and Gli2ΔN were gifts of Andrzej Dlugosz (University of Michigan). 293T cells were plated in 12-well plates, transfected with 0.7 μg/well transcription factor, 0.4 μg/well reporter plasmid, and 2 ng/well pRL-TK Renilla (Promega), and analyzed for luciferase expression 36 h after transfection using the Dual-Luciferase Reporter kit (Promega) following the manufacturer's protocol. Firefly luciferase expression was normalized by well to Renilla, and fold changes were calculated by comparing to 8xGli-Luciferase transfected alone. Statistical analysis was performed using the Student's *t*-test. For immunofluorescence, 293T cells grown on collagen-coated coverslips were transfected with 1.5 μg/well of transcription factor and analyzed after 36 h. FLAG-tagged GLI1 isoforms were detected with mouse-anti-FLAG (Sigma) and anti-mouse AlexaFlour488 (Molecular Probes) both at 1:1,000.

### Cytokine Expression QPCR

Colonic tissue was carefully cleaned of all fatty tissue and whole colonic mRNA was collected using Trizol, followed by RNA clean-up with DNase digestion using the RNeasy Mini Kit (Qiagen). cDNA was synthesized using the iScript cDNA synthesis kit (Biorad), and SybrGreen QPCR was performed on a Biorad iCycler. Expression levels were normalized to GAPDH, and statistical analysis was performed using the Student's *t*-test.

### Statistical Analysis

Haplotype frequencies of the tSNPs were inferred using the expectation-maximization algorithm and these used to test whether haplotype frequencies were different in cases and controls as implemented in the EH and PM programmes. The test statistic 2 × (lnLcase] + ln[Lcontrol] – ln[Lcase/Lcontrol]), which has a χ^2^ distribution with *n* − 1 degrees of freedom (where *n* = number of possible haplotypes) was calculated and empirical *p*-values obtained by permuting the data 10,000 times. Haplotypes were examined and linkage disequilibrium between individual SNPs (*r* ^2^) calculated using the Haploview programme v3.2 (http://www.hapmap.org). Individual SNP analysis was performed using χ^2^ or Fisher's exact test, where appropriate, with two-tailed *p*-values given and odds ratios (OR) presented with 95% confidence intervals (CIs). The meta-analysis of SNP rs2228226 was performed using the Mantel-Haenszel method using a fixed effects model (R-software package). Details for calculation of false positive report probability (FPRP) are provided in Supporting Information (Text S1). Expression profiles were analysed using Mann-Whitney *U*-test and Kruskal-Wallis test, assuming a nonparametric distribution of all datasets (GraphPad Prism 4, GraphPad Software Inc.).

## Results

### Gene-Wide Variation in *GLI1* Is Associated with IBD and Attributable to a Nonsynonymous SNP (rs2228226) in the Scottish Population

Four multi-marker tSNPs (*r* ^2^ ≥ 0.8) were identified (rs3817474, rs2228225, rs2228224, and rs2228226) to describe haplotypic variation of *GLI1*, detecting haplotypes of a frequency >1%. We genotyped these four tSNPs in a Scottish IBD population consisting of 474 UC and 335 CD cases, and 1,364 well-matched HCs (Table 1). We then used a model-free analysis [30] to test the association of *GLI1* and IBD susceptibility. In the Scottish population, we demonstrate a highly significant association in IBD (*p* < 0.0001) and UC (*p* < 0.0001), and an association with CD of borderline significance (*p* = 0.03). On analysis of individual estimated haplotype frequencies in Haploview, three common haplotypes were described (A to C; Table S1). We confirmed that this effect was confined to the *GLI1* gene by genotyping an additional seven haplotype-tagging SNPs, chosen from Phase II HapMap data, to tag neighbouring blocks, in 166 CD and 170 UC patients. This confirmed the presence of a *GLI1* spanning haplotype block that did not extend into neighbouring genes (*INHBE* and *ARHGAP9*) (Figure S2).

The association on haplotype testing and log-likelihood analysis was largely attributable to a nonsynonymous SNP in exon 12 of *GLI1* (rs2228226C→G; tSNP_4_). rs2228226 was associated with IBD (allelic frequency OR = 1.23, CI 1.07–1.40, *p* = 0.0026; homozygotes OR = 1.56, CI 1.15–2.11, *p* = 0.0047), CD (allelic frequency OR = 1.30, CI 1.08–1.55, *p* = 0.0053, homozygotes OR = 1.79, CI 1.21–2.65, *p* = 0.0048), and UC (allelic frequency OR = 1.19, CI 1.01–1.39, *p* = 0.04; homozygotes OR = 1.41, CI 0.98–2.03, *p* = 0.079) (Tables 2 and S2). These data suggest an allele specific dose response with a greater OR for homozygotes than heterozygote patients. Mutation screening of the *GLI1* coding regions by direct sequencing failed to identify any novel SNPs. There was no association between seven additional *GLI1* variants from dbSNP and IBD (Table S2). Despite tight linkage disequilibrium (LD) across *GLI1* (Figure S2), there was notably less LD between rs2228226 and other tSNPs (*r* ^2^ = 0.7) in both cases and controls (Figure S3), and this finding is likely to provide the explanation for the lack of association in neighbouring SNPs.

**Table 2:**
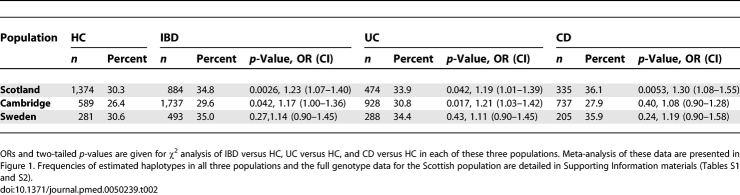
Minor Allelic Frequencies for *GLI1* Nonsynonymous SNP rs2228226 (tSNP4) in Scottish, English, and Swedish HC, IBD, CD, and UC

**Figure 1:**
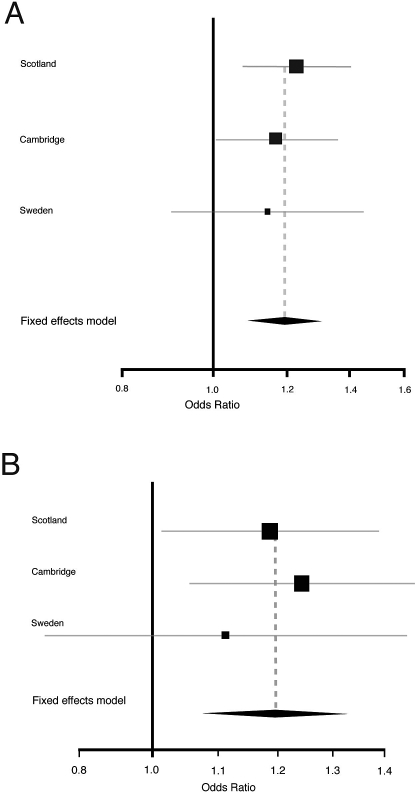
Meta-Analysis of Nonsynonymous *GLI1* SNP rs2228226 (tSNP_4_) in Scotland, Cambridge, and Sweden Using Mantel-Haenszel Method (*n* = 5,352 Individuals) (A) IBD versus HCs. (B) UC versus HCs. There was no evidence of heterogeneity in the contribution of rs2228226 between the three cohorts (*p* = 0.825). 95% CIs for individual populations are represented by horizontal lines and population sizes by square boxes. The diamond represents the pooled OR (fixed effect model) with 95% CIs delineated by the diamond's width. Note the different range of the *x*-axis for (A) and (B).

### Replication of *GLI1* Association in Two Independent Northern European IBD Cohorts and Meta-Analysis

We then sought to replicate these findings in other populations. In a large IBD panel from Cambridge, England (*n* = 928 UC, 737 CD, 83 IBDU, and 589 HC) association with *GLI1* was replicated by log-likelihood analysis in IBD (*p* = 0.009) and UC (*p* < 0.0001). rs2228226 was associated with IBD (OR 1.17, CI 1.00–1.36, *p* = 0.042) and UC (OR 1.21, CI 1.03–1.42, *p* = 0.017), but not CD in this population (Table 2). As in Scotland, there was no association with tSNPs_1−3_. Minor allelic frequencies differed slightly from the Scottish population; this difference is in keeping with that noted for a number of SNPs analysed for population stratification in the WTCCC study [3]. In the smaller Swedish cohort (*n* = 770), there was a consistent trend to association of rs2228226 with IBD (OR 1.14, CI 0.90–1.45) (Table 2) although this association was not statistically significant. However, the allelic frequencies in cases and controls were very similar in Scotland and Sweden indicating that the lack of statistical significance in the latter cohort is due to insufficient statistical power. There were no consistent genotype–phenotype associations across the populations, including stratification for colonic IBD.

A meta-analysis of Scottish, English, and Swedish data for rs2228226, using the Mantel-Haenszel method with a fixed effects model on IBD versus HCs (OR 1.194, CI 1.089–1.309, *p* = 0.0002) (Figure 1A) confirmed the association of this variant in Northern Europe, achieving criteria for significance in a gene-centric study [31,32]. The meta-analysis was repeated separately for UC cases versus HCs with a similar effect size noted (OR 1.196, CI 1.077–1.327, *p* = 0.0008) (Figure 1B).

Recognising the current problem with the publication of false positive findings in genetic association studies we estimated the probability that the association with disease risk found in the meta-analysis of *GLI1* SNP rs2228226 represents a true (rather than false positive) association by adopting the FPRP approach described by Wacholder [33]. This gives an estimated probability that these findings represent a true finding of at least 92% (FPRP < 0.08). This method is designed to avoid over-interpretation of statistically significant findings that are not likely to signify a true positive. In our study, this parameter gives clear support to our interpretation of these data.

### The GLI1 Variant Encoded by rs2228226 Is Functionally Deficient in Activating GLI-Responsive Transcription In Vitro

**Figure 2:**
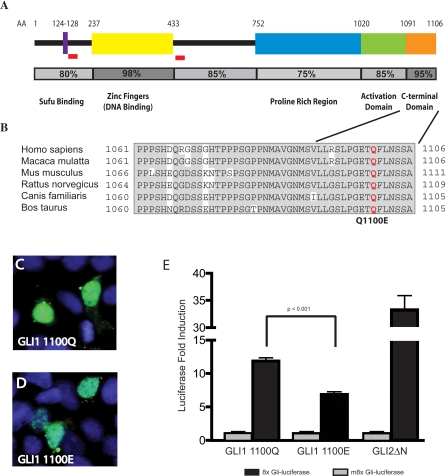
Q1100E Disrupts a Conserved Region of the GLI1 Protein and Reduces GLI1 Transcriptional Activity (A) Conservation of known functional domains in the Gli1 protein. Previously described Sufu binding, DNA binding, and transactivation domains [34,57,58] are shown schematically. Amino acid conservation of each domain is represented numerically and by shading of the bar below the domain. Red boxes indicate regions known to regulate GLI1 protein stability [49]. The conserved C-terminal domain that includes Q1100E is adjacent to a known transactivation domain. (B) Alignment of the C terminus of mammalian Gli1 proteins. This region (AA 1080–1106) is highly conserved in mammalian lineages. (C, D) GLI1 Q1100 and E1100 have similar cellular localization in 293T cells. (E) GLI1 E1100 is deficient in driving activation of the 8xGli-Luciferase reporter compared to GLI1 Q1100. GLI2ΔN is a strong activator of 8xGli-Luciferase and serves as a positive control for GLI1 activation. The m8xGli-Luciferase construct contains eight mutant Gli binding sites and serves as a negative control. Data are shown from six triplicate experiments done using two different plasmid preparations (*n* = 18). Bar heights indicate mean fold activation above baseline, and error bars indicate standard error of the mean. *p*-Values were calculated using the Student's *t*-test.

rs2228226C→G is a missense mutation in exon 12 of *GLI1*, encoding a change from glutamine to glutamic acid (Q1100E). The mutation falls within a well conserved motif at the C terminus of mammalian GLI1 proteins, near a recognized transactivation domain (Figure 2A) [34]. The Q1100 residue is itself 100% conserved in all mammals examined (Figure 2B). In order to evaluate the functional consequences of the Q1100E mutation, we transfected either GLI1 Q1100 or the variant GLI1 E1100 into 293T cells. No significant differences in level of expression or cellular localization were detected between these GLI1 variants; both proteins were readily detectable in the nucleus of transfected cells (Figure 2C and 2D). Blinded quantitation of cellular localization demonstrated a range of cytoplasmic and nuclear localization that was similar for both GLI1 variants (unpublished data). We further evaluated the ability of each variant to activate the well-characterized GLI reporter 8xGli-Luciferase [35]. We utilized Gli2ΔN, a very strong activator of 8xGli-Luciferase, as a positive control for GLI1 transcriptional activity [36]. While both GLI1 variants activated 8xGli-Luciferase above baseline, WT GLI1 Q1100 was 2-fold more efficient as a transcriptional activator than the variant GLI1 E1100 (Figure 2E).

### HH Pathway Activity Is Dysregulated in Colonic Inflammation

**Figure 3:**
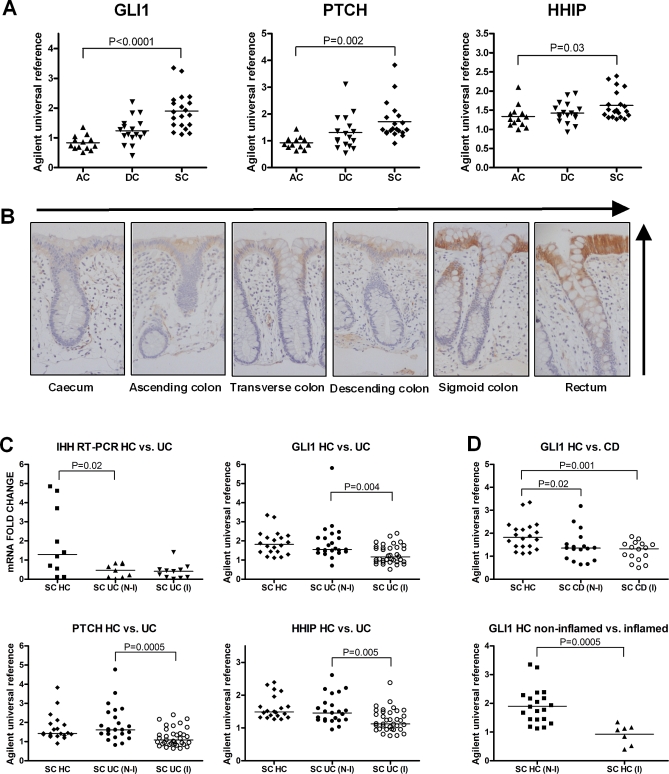
Expression of HH Signalling Components in the Healthy Human Adult Colon (HC) and UC (A) PTCH, HHIP, and GLI1 mRNA levels increase along the length of the healthy adult colon, from ascending colon (AC) to descending colon (DC) and sigmoid colon (SC). (B) HH protein expression in terminally differentiated enterocytes at the luminal surface, is greater in the distal colon compared with the proximal (vertical and horizontal arrows represent these gradients). (C) Quantitative analysis of mRNA levels of IHH, PTCH, GLI1, and HHIP in UC compared with noninflamed HC (HC N-I). To account for the gradients identified along the length of the healthy colon (a-b), the data from SC only are shown. QPCR data is presented for IHH as this gene was not present on the Agilent microarray chip. (D) GLI1 expression in CD versus HC (N-I) and non-IBD inflammation (HC I) versus HC. Disease specimens are subcategorised into noninflamed (N-I) and inflamed (I) tissues. There was no change in levels of desert HH (DHH), PTCH2, GLI2, GLI3, SUFU, or DISP1 in either UC or CD compared with HC, or in non-IBD inflammation (unpublished data). Analysis of SHH mRNA demonstrated a mild increase in expression levels related to inflammation that is consistent with the known expression of SHH in inflammatory cells (Figure S4) [21]. Individual data points are plotted with horizontal lines representing the medians for each dataset. *p*-Values presented are derived from Kruskal-Wallis test, comparing levels in AC, DC, and SC, and from Mann-Whitney *U*-tests (UC versus HC (N-I)).

Extended in silico analysis of microarray data demonstrates that mRNA transcripts of PTCH and hedgehog-interacting protein (HHIP), along with HH protein, are greater in the distal compared with the proximal colon in humans, mirroring the expression gradient of GLI1 previously reported [26] (Figure 3A and 3B). GLI1, PTCH, and HHIP are pathway response elements whose expression levels predict pathway activity [14]. GLI1 (*p* = 0.0003), PTCH (*p* = 0.002), and HHIP (*p* = 0.0003) were lower in inflamed UC compared with HC from equivalent location (Figure 3C). IHH was lower in UC regardless of inflammation (*p* = 0.02). GLI1 expression was lower in CD than HC (*p* = 0.004) irrespective of inflammatory status (Figure 3D), a noteworthy finding given that *GLI1* variation was also associated with CD in Scotland. PTCH was lower in inflamed CD compared with noninflamed CD and HC (*p* = 0.007) (Figure S5). GLI1 and PTCH were both lower in non-IBD inflammation versus HC (Figures 3D and Figure S6). Treatment for IBD (5-amino salicylic acid, thiopurines, and corticosteroids) did not influence expression of GLI1 (Figure S7). These data demonstrate overall down-regulation of HH pathway activity, including GLI1, PTCH, and HHIP, in areas of colonic inflammation.

### *Gli1^+/lacZ^* Animals Exhibit Mortality and Heightened Morbidity in Response to Intestinal Inflammation Induced by 3% DSS Treatment

In vitro analysis of the GLI1 1100E variant demonstrated a 50% deficiency in transactivation function compared to WT GLI1 and our genetic analysis demonstrated an allele-specific dosage response, suggesting that a moderate reduction in GLI1 function may predispose to intestinal inflammatory disease. To specifically test this hypothesis, we treated *Gli1^+/lacZ^* mice [37] and their WT littermates with 3% DSS to induce acute intestinal inflammation.

**Figure 4:**
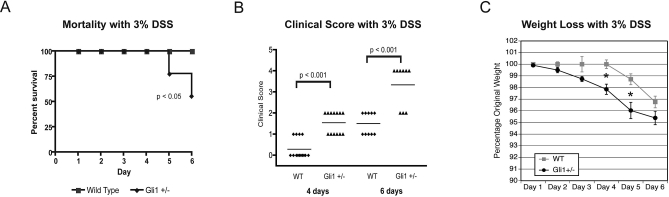
*Gli1^+/lacZ^* Animals Show Mortality, Severe Clinical Symptoms, and Profound Weight Loss after DSS Treatment (A) Kaplan-Meier survival curve. WT animals are 100% viable over the 6-d treatment period (*n* = 14). Nearly 50% of *Gli1^+/lacZ^* animals (four out of nine) die in response to 3% DSS treatment for 6 d. (B) *Gli1^+/lacZ^* animals display markedly more severe symptoms than WT animals after 4 or 6 d of 3% DSS treatment. 1, diarrhoea; 2, bloody diarrhoea; 4, severe bleeding/death. Each dot represents an individual animal and the solid line shows the mean observation in each cohort. (C) *Gli1^+/lacZ^* animals (*n* = 9) lose weight more rapidly than their WT littermates (*n* = 10). *, *p* < 0.05 by Student's *t*-test.

*Gli1^+/lacZ^* animals were rapidly and severely affected by DSS treatment. After 6 d, four out of nine had died, and three of the survivors demonstrated severe morbidity, with significant rectal bleeding and almost complete immobility (Figure 4A). In contrast, no WT animals (*n* = 14) died, all were mobile and showed less morbidity on days 5 and 6 after treatment. *Gli1^+/lacZ^* animals developed bloody diarrhoea and significant weight loss by day 4, whereas WT animals did not develop clinical signs or measurable weight loss until day 6 (Figure 4B and 4C). The rapid onset of weight loss, clinical signs, and mortality in *Gli1^+/lacZ^* animals was potentially indicative of extensive colonic inflammation, leading us to directly assess the histology of *Gli1^+/lacZ^* and WT colons after DSS administration.

### *Gli1^+/lacZ^* Animals Develop More Severe Colonic Pathology Than WT Littermates in Response to DSS Treatment

**Figure 5:**
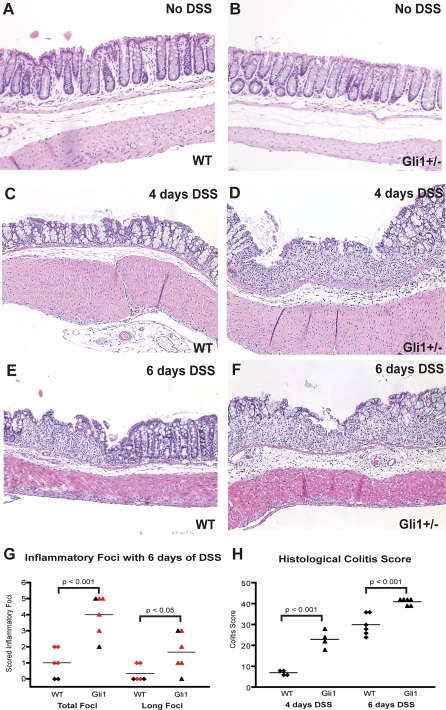
*Gli1^+/lacZ^* Animals Demonstrate More Severe Intestinal Inflammation than WT Littermates in Response to DSS Treatment (A, B) WT and *Gli1^+/lacZ^* animals demonstrate normal thickness and structure of colonic mucosa in the absence of DSS. (C) WT animals (*n* = 4) exhibit mild colonic inflammation but do not develop substantial epithelial or ulcerative inflammatory pathology within 4 d of DSS treatment. (D) *Gli1^+/lacZ^* animals (*n* = 4) develop significant inflammatory infiltration, epithelial damage, and ulceration within 4 d of DSS treatment. (E) WT animals (*n* = 14) demonstrate ulceration following DSS treatment. (F) *Gli1^+/lacZ^* animals develop profound intestinal inflammation in response to 3% DSS treatment, with severe epithelial damage in long stretches of their colonic mucosa (*n* = 9). (G) Blinded histological scoring of colonic damage after 6 d of DSS treatment. Standard lengths of tissue from the mid colon and distal descending colon were scored in each animal. *Gli1^+/lacZ^* animals (*n* = 6) have more overall inflammatory foci and more long foci (10+ crypt units affected) than WT animals (*n* = 6). Each dot represents the number of observed foci in an individual animal; the solid line shows the mean observation in each cohort. Red dots indicate the animals that were analyzed for cytokine expression. (H) Comprehensive colitis scoring [29] of WT and *Gli1^+/lacZ^* animals after 4 and 6 d of DSS treatment. *p*-Values for (G) and (H) calculated by the Student's *t*-test.

Untreated WT and *Gli1* ^+/lacZ^ animals had intact colonic mucosa; no clinical or histological signs of inflammatory disease were present prior to DSS treatment (Figure 5A and 5B). After 4 d of DSS treatment, WT colons exhibited evidence of inflammatory change but with few destructive lesions (Figure 5C), while extensive inflammatory infiltration and destructive colonic ulcers were prominent in *Gli1^+/lacZ^* mice (Figure 5D). After 6 d, the number, size, and invasiveness of inflammatory lesions (Figure 5E–5G) were significantly greater in *Gli1^+/lacZ^* animals, and the overall colitis score (Figure 5H) [29] was significantly greater. Taken together, these results demonstrate that the loss of a single *Gli1* allele leads to increased sensitivity to DSS treatment as reflected by severe intestinal inflammatory pathology and clinical signs.

### Intestinal Myeloid Cells Respond Directly to HH Signals

**Figure 6:**
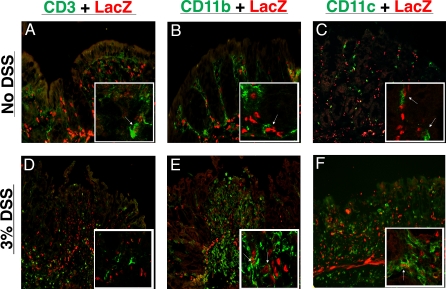
Intestinal Myeloid Cells Respond Directly to Hh Signalling during Homeostasis and Inflammation (A–C) No DSS treatment. (D–F) 4 d of 3% DSS. (A, D) Few CD3+ T lymphocytes respond to Hh signalling during colonic homeostasis. Infiltrating T cells in DSS-induced ulcers do not express Gli1 or respond to Hh signalling. (B, E) Many but not all CD11b+ myeloid cells respond to Hh signalling and express LacZ. After 4 d of DSS, ulcers in *Gli1^+/lacZ^* animals are filled with myeloid cells, many of which respond to Hh signals. (C, F) CD11c+ dendritic cells respond directly to Hh signals during homeostasis and inflammation. Nuclear DAPI staining was performed but has been omitted to allow clear visualization of co-expression of nuclear LacZ and cell surface markers.

We previously demonstrated that HH signalling is paracrine in murine intestine and colon [38]. Given that *Gli1^+/lacZ^* animals contain a copy of bacterial β-galactosidase under the control of the *Gli1* promoter, they represent an excellent model to confirm this observation and identify cell types that are Hh-responsive (i.e., LacZ positive) before and during inflammatory disease. These studies revealed that no autocrine Hh signalling was detectable in untreated adult colon or following inflammatory stress; colonic epithelial cells do not express Gli1 or respond to Hh signals during colonic homeostasis or in areas of either mild or severe epithelial perturbation (Figure 6 and unpublished data). We did, however, detect a large population of Hh-responsive cells within the inflammatory infiltrate of DSS-induced ulcers after 4 d of DSS treatment in *Gli1^+/lacZ^* animals (Figure 6D–6F). Both lymphocytes [21,22] and myeloid cells [23] have recently been shown to respond to Hh signals outside of the gastrointestinal tract. We therefore investigated whether these populations were responding in the adult colon. We detected CD3-positive T lymphocytes, CD11b-positive myeloid cells, and CD11c-positive dendritic cells responding to Hh signalling in resting colon without DSS treatment (Figure 6A–6C). After 4 d of DSS treatment, significant numbers of Hh-responsive myeloid and dendritic cells were seen in inflammatory ulcers (Figure 6E and 6F). We did not detect Hh response amongst the infiltrating T cells in DSS-induced ulcers (Figure 6D). Examination of CD19-postive B lymphocytes in resting colon and after both 4 and 6 d of 3% DSS treatment revealed no clear Hh response in this population (unpublished data). Taken together, these data suggest that myeloid cells, including dendritic cells, are direct targets of Hh signalling in the colon both during homeostasis and after inflammatory stress. This finding, in association with the findings above, implies that direct Hh signalling may play a role in innate immune function in the colon.

### *Gli1^+/lacZ^* Animals Have Dramatically Increased IL-23p19 and Pro-inflammatory Cytokine Expression after DSS Treatment

**Figure 7:**
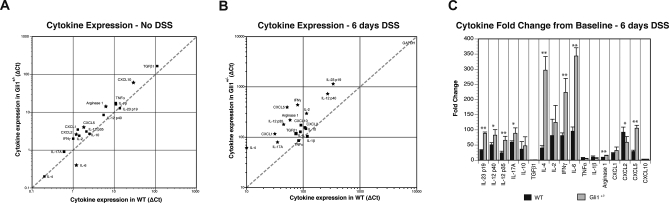
Cytokine Analysis of *Gli1^+/lacZ^* and WT Mice after DSS Treatments Demonstrates Robust Pro-inflammatory Cytokine Activation (A, B) Cytokine and chemokine expression normalized to GAPDH is plotted on the *y*-axis for *Gli1^+/lacZ^* mice, and on the *x*-axis for WT animals. Gene expression levels that are statistically different are shown with stars. The dotted diagonal trend line indicates identical expression levels between WT and *Gli1^+/lacZ^* mice. (A) Cytokine and chemokine expression in WT and *Gli1^+/lacZ^* animals (*n* = 3) without DSS treatment. Cytokine and chemokine expression is low and very similar between WT and *Gli1^+/lacZ^* animals. (B) Cytokine expression in WT and *Gli1^+/lacZ^* animals (*n* = 4) after 6 d of 3% DSS treatment. Many pro-inflammatory cytokines and chemokines are significantly more highly expressed in *Gli1^+/lacZ^* animals compared to WT. Note the difference in axes in panels (A) and (B). (C) Fold change of cytokine expression from baseline to inflamed for WT and *Gli1^+/lacZ^* animals. We detect dramatic up-regulation of T_h_17 and T_h_1 pathway cytokines and chemokines in *Gli1^+/lacZ^* animals. *, *p* < 0.05; **, *p* < 0.01 by the Student's *t*-test.

Overall, unchallenged WT and *Gli1^+/lacZ^* animals have a similar cytokine and chemokine profile as determined by QPCR examination of whole colonic tissue (Figure 7A). There are, however, several molecules (Cxcl2, Cxcl5, Cxcl10, and Arg1) that are modestly increased in *Gli1^+/lacZ^* animals, and a small decrease in IL-6 expression; these changes may indicate a subtle difference in the inflammatory milieu of *Gli1^+/lacZ^* animals at baseline. More strikingly, after 6 d of DSS *Gli1^+/lacZ^* animals demonstrate robustly up-regulated expression of several pro-inflammatory cytokines and chemokines compared to WT (Figure 7B). When baseline expression values are compared to values after DSS challenge for each genotype (Figure 7C), robust expression and up-regulation of T_H_1 cytokines, including IFNγ, is detectable in *Gli1^+/lacZ^* animals (Figure 7B and 7C). We did not detect a significant difference in TGFβ and IL-10 between *Gli1^+/lacZ^* and WT animals, suggesting that down-regulation of anti-inflammatory cytokines was not the primary mechanism of increased inflammation in this model.

The most highly expressed cytokine after DSS treatment in *Gli1^+/lacZ^* animals was IL-23p19, a molecule that drives differentiation of T_H_17 lymphocytes, key mediators of inflammation in several systems, including IBD [39,40]. IL-12 and IL-17, cytokines closely associated with IL-23, were also robustly up-regulated in *Gli1^+/lacZ^* animals. In fact, this entire pathway was highly up-regulated from the baseline in *Gli1^+/lacZ^* animals compared to WT littermates (Figure 7B and 7C). These findings are particularly interesting since the IL-23 pathway has recently been strongly implicated in IBD pathogenesis both in humans [4] and mice [40]. Taken together, these cytokine data confirm that animals lacking one genomic copy of *Gli1* develop significantly increased inflammatory disease compared to WT littermates in response to the same inflammatory challenge.

## Discussion

The data presented here provide the first evidence, to our knowledge, that intact HH signalling is critical in the mammalian gut response to inflammatory challenge, and suggest that reduced GLI1 function is implicated in IBD pathogenesis. We confirm that the HH signalling pathway is down-regulated in colonic inflammation in humans. We identify a specific *GLI1* variant that is highly associated with UC/IBD, and demonstrate that the variant protein is functionally deficient as a transcriptional activator in vitro. Finally, we demonstrate that mice with a 50% reduction in *Gli1* exhibit a heightened intestinal inflammatory response to DSS with significant up-regulation of the IL-23 pathway. Not only do these findings have clear implications for the understanding of IBD pathogenesis and suggest potential for therapeutic intervention, they are the first clear description of a functional role for HH signalling and GLI1 in bowel inflammation.

The inherited variation in the *GLI1* gene that we have detected is associated with IBD and UC, in both Scotland and England, with findings for rs2228226 confirmed by meta-analysis of over 5,000 individuals, with OR of 1.19. Evidence for an effect in CD is seen in the present study, but the predominant effect is clearly related to UC, consistent with the reported IBD2 linkage studies [11,13]. The magnitude of this association is entirely in line with the effect size noted in a number of recent studies of complex disease genetics, including CD [41], colo-rectal cancer [42], and coeliac disease [43]. The level of significance attained satisfies suggested criteria of *p* < 10^−4^ to 10^−6^ for gene-centric studies [31,32]. Population heterogeneity has been previously described at the IBD2 linkage region [11,13,44,45]. The three Northern European populations studied here have previously demonstrated similar contribution of other IBD susceptibility genes/loci, including NOD2 and IBD5 [46].

Whilst our resequencing efforts identify rs2228226 as the only coding variant associated with IBD, further deep resequencing will be important in the future as the haplotype analysis and log-likelihood analyses raise the possibility that other germ-line variants may also contribute to IBD risk. These need be explored formally—specifically the role of intronic variants, long-range promoter effects, and/or copy number variation. In this context, several complex disease genes, including NOD2 [8,9], have multiple independent mutations conferring disease risk, some disease genes have no causative mutations within coding sequences (e.g., *IRGM* in CD [10,47]), and synonymous SNPs may be associated with functional effects [48].

rs2228226C→G encodes a change from glutamine to glutamic acid (Q1100E). Our in vitro data demonstrate that GLI1 1100E is a subfunctional transcriptional activator compared to WT GLI1, though it is appropriately synthesized and localized. The Q1100E mutation causes a significant charge change in a conserved region directly adjacent to the known transactivation region of GLI1; this change could directly modify transactivation activity, disrupt the structure of the transactivation domain, or affect protein stabilization [49], decreasing activity. Our murine data confirm that reduction in GLI1 activity has significant consequences for the inflammatory response.

Intestinal HH signalling in mice and humans is exclusively paracrine: HH ligands in the epithelium signal to the HH response network (PTCH, GLI1, HHIP) in the mesenchyme [38,50,51]. This has been demonstrated during development [38], homeostasis (Kolterud A, Grosse AS, Zacharias WJ, Walton KD, and Gumucio DL, personal correspondence), malignancy [51], and now, in this report, in inflammation. The human expression data presented here also support the relevance of the HH pathway to colonic inflammation in humans. GLI1 expression, a reliable indicator of pathway activity, is down-regulated in all forms of colonic inflammation examined (UC, CD, and non-IBD inflammation). Whilst embryonic HH pathway activity is reportedly recapitulated in diverse organs/tissues in response to acute injury and inflammatory challenge [15–19], GLI1 expression is notably decreased in psoriasis, a disease of the epithelial border that shares some pathogenic features with IBD [52].

The localisation of HH protein demonstrated here is similar to that shown for IHH by van den Brink and colleagues by in situ hybridisation [53]. However, along with other commentators, we would urge caution in the use of the commercially available HH pathway antibodies presently available [54]. We note further that the expression analysis of disease samples in humans is inherently limited in that it only depicts a complex process at a late time point in established disease. Nonetheless, in combination with studies utilizing models (such as the *Gli1^+/lacZ^* mouse) to directly correlate HH response with inflammatory challenge, these studies provide useful information to help elucidate the aetiology of human inflammatory disease.

*Gli1^+/lacZ^* animals, which have only 50% of the WT level of Gli1, develop severe and rapid inflammation in response to DSS, despite having normal histological structure and a largely WT cytokine and chemokine profile prior to DSS treatment. These data, to our knowledge, provide the first description of a phenotype for reduced Gli1 function [37], and demonstrate the key role that a full complement of Gli1 plays in protection from inflammatory disease. Our in vitro data demonstrate that GLI1 E1100 is capable of activating some GLI1 response, suggesting that under homeostatic conditions, GLI1 E1100 could perform adequately. Similar to the situation in *Gli1^+/lacZ^* animals, however, under conditions of inflammatory stress, GLI1 E1100 can only function at 50% of the level of WT GLI1. Whether the predisposition to inflammation in these systems is a direct result of lowered HH signal transduction within HH-responsive inflammatory cells or reflects the effect of lower HH signals on other stromal target cells [15] that, in turn, release signals that impact the integrity of the epithelial layer or the inflammatory phenotype of resident immune cells is not yet clear, and will be an important area of future study. Furthermore, we believe that it will be important to determine if other primary (e.g., germ-line variation in other HH pathway genes and the HH interactome, epigenetic changes, and gene–environmental interactions) or secondary phenomena (e.g., due to alterations in commensal flora) in the human/murine intestine alter the transduction of HH signals in response to acute and chronic inflammatory challenge.

We have additionally shown that the HH pathway may directly modify the innate immune response through signalling to myeloid target cells. This finding is in accord with recent data demonstrating a crucial role for Hh signalling in myeloid cell maturation in the spleen [23]. Interestingly, myeloid cell populations are known to modify the intestinal inflammatory milieu through a significant impact on the IL-23/IL-17 pathway [55], a pathway that we find to be clearly and distinctly dysregulated in *Gli1^+/lacZ^* inflammation. Taken together, these data raise the exciting possibility that the normal role of HH signalling is to promote a tolerogenic phenotype in mucosal myeloid cells. In the face of reduced HH signal transduction in these cells, minor inflammatory stimuli may trigger robust inflammatory responses. Future studies directly investigating this hypothesis by specifically testing the effects of Hh signalling on intestinal myeloid cells will be crucial to the evolution of a more complete understanding the role of Hh in intestinal inflammation.

### Limitations of the Study

A number of issues meriting further work emerge from the present study. Firstly, deep resequencing of the entire *GLI1* gene is required to identify additional variants that contribute to the overall disease association noted for the gene. Secondly, the in vivo examination of reduced Gli1 function is presently limited to heterozygous mice. Despite the robust phenotype observed in these mice following acute inflammatory challenge, these findings need to be extended to homozygous null mutant animals (*Gli1* ^−/−^). Thirdly, additional animal models of colitis, particularly of chronic disease, need to be examined; for example, trinitrobenzene sulfonic acid (TNBS) induced colitis, or adoptive transfer of CD4^+^CD45RB^high^ T cells into recombinase-activating gene (*rag*)-1 deficient (*rag1* ^−/−^) mice. Finally, we have not yet directly examined the phenotype of reduced GLI1 function in antigen presenting cells in vitro.

### Conclusions

A key paradigm emerging in complex disease genetics is that gene discovery may lead to fundamental shifts in the direction of basic and translational research efforts. Recently, it is apparent that mechanistic insights may have greater significance than the index gene discovery itself, especially when one considers the small contribution each locus/variant makes towards disease susceptibility. IBD has provided several recent examples. Notably, the discoveries of *NOD2* and *IL-23R* have launched academic and pharmaceutical research efforts into innate immunity and Th17 signalling, whereas *ATG16L1* and *IRGM* have focused much attention on the role of autophagy in CD pathogenesis. In the present study, our genetic studies led in turn to detailed observations of expression in established human disease as well as a mechanistic study in the mouse. We believe that these combined findings have the potential to open entirely novel lines of enquiry that may have great consequences for the understanding of IBD pathogenesis and potentially for inflammatory pathways in other HH-expressing organs (e.g., skin, lung, bone).

In conclusion, we demonstrate here the dysregulation of a developmental signalling pathway in IBD for the first time to our knowledge. Furthermore, we show that these effects are in part genetically determined, with evidence implicating *GLI1* as an IBD2 gene, and identification of a specific variant with reduced transcriptional activity. The functional relevance of *Gli1* is demonstrated by the severe intestinal inflammation that develops in the face of a 50% reduction in Gli1 concentration in an established mouse model of colitis. Taken together, these data strongly argue that tolerance to inflammatory stimuli requires a fully functional HH signal transduction network. With HH signalling modulators under active investigation in a number of clinical trials (http://www.curis.com and http://www.clinicaltrials.gov), these data provide promise for the development of novel therapeutic strategies. Since the use of HH inhibitors has recently been advocated for the treatment of certain tumours that secrete HH, our finding that reduced HH signalling has inflammatory consequences suggests that inflammatory status should be carefully followed during upcoming Phase II trials [56].

## Supporting Information

Figure S1 Schematic of Activation Events in the HH Signalling Pathway There are three mammalian homologues of *Drosophila* gene *Hh*, of which SHH and IHH have important functions in the gut and the immune system. Intestinal HH expression is limited to epithelial cells, while HH-response genes are expressed only in intestinal mesenchyme. In epithelial cells, soluble HH is processed in the Golgi and released via Dispatched (DISP1). In the mesenchyme, upon HH binding to the transmembrane receptor PTCH, PTCH can no longer repress SMO, and SMO prevents cleavage of transcription factors GLI2 and GLI3 from activator to repressor form; GLI activator forms then predominate, and transcription is activated in a raft of downstream targets. Direct early targets of HH signals include GLI1, a strong transcriptional activator which acts an amplifier of HH signalling, PTCH, and HHIP, a feedback inhibitor; these markers can be used as indicators of pathway activity [14,54].
(66 KB PPT)

Figure S2 The Haplotype Structure in *GLI1* and Surrounding Haplotype Blocks Are Presented in Haploview from Genotyping in UC (A) and CD (B) There is a haplotype block spanning the *GLI1* gene that does not extend into neighbouring genes. LD is described here by D′ values.
(1 MB TIF)

Figure S3 The Haplotype Structure of the Four *GLI1* tSNPs from the Scottish HCs and UC Population and from HapMap CEU Data Is Demonstrated with *r* ^2^ Values The LD between rs2228226 and neighbouring SNPs is overestimated by D′ (Figure S2). The *r* ^2^ value between rs2228226 (tSNP_4_) and rs2228224 (tSNP_3_) in UC is 0.7, providing an explanation for the evidence of association at rs2228226 in the absence of association at neighbouring SNPs.
(325 KB TIF)

Figure S4 Quantitative Analysis of mRNA Levels by Microarray and RT-PCR of SHH in UC Compared with Noninflamed HCs For the microarray data on the left, disease specimens are taken from the ascending colon (AC), descending colon (DC), and sigmoid colon (SC). To take into account anatomical gradients of HH signalling in Figure 3, RT-PCR (right) is performed on samples from SC only, and UC samples are subcategorised into noninflamed (N-I) and inflamed (I). This observation validates the microarray data and shows that the modest increase in SHH is present only with inflammation. This result is of interest given the fact that inflammatory cells have been shown to express SHH. *p*-Values represent Kruskal-Wallis analysis of HC versus UC for each anatomical location.
(1.9 MB TIF)

Figure S5 Quantitative Analysis of mRNA Levels by Microarray and RT-PCR of SHH, GLI1, PTCH, and HHIP in CD Compared with Noninflamed HCs Disease specimens are taken from the terminal ileum (TI), ascending colon (AC), and sigmoid colon (SC), and are subcategorised into noninflamed (N-I) and inflamed (I) tissues. *p*-Values represent Kruskal-Wallis analysis of HC, CD (N-I), and CD (I) for each anatomical location.
(1.8 MB JPG)

Figure S6 Analysis of SHH, PTCH, and HHIP mRNA Levels by Microarray in Colonoscopic Biopsies from the Sigmoid Colon (SC) of Non-IBD patients with Inflammation (I) Compared with Noninflamed HCs (NI) (1.7 MB JPG)

Figure S7 Analysis of GLI1 Expression in UC by Treatment Data are presented for UC patients on no treatment (Rx), on a 5-amino salicylic acid (5-ASA) only, on azathioprine (AZA), or mercaptopurine (MP) with or without a 5-ASA and on steroids (oral and topical) with or without a 5-ASA. Individual data points are represented with horizontal lines representing medians. There was no difference between any of the groups on analysis by Mann-Whitney *U*-testing.
(932 KB JPG)

Figure S8 The Specificity of the N-19 HH Antibody Is Demonstrated with Application of the Primary Antibody, Primary Antibody + SHH Blocking Peptide Pre-absorbed Overnight, and with Primary Antibody Omitted This figure demonstrates that the N-19 HH antibody reacts with the peptide but does not preclude it from cross-reacting with other proteins. Indeed, we would agree with other commentators [54] in urging caution in the use of this and other presently available HH antibodies for immunohistochemistry.
(289 KB TIF)

Table S1 *GLI1* Haplotype Frequencies in IBD, UC, CD, and HC in (A) Scotland, (B) Cambridge, England, and (C) Sweden tSNP_1_, rs3817474; tSNP_2_, rs2228225; tSNP3, rs2228224; tSNP_4_, rs2228226. Differences between IBD, CD, UC, and HC frequencies are shown, calculated by χ^2^ test, with two-sided *p*-value, OR, and 95% CIs. Estimated haplotype frequencies were calculated using Haploview, version 3.2. Log-likelihood *p*-values (calculated on the PM/EH platform with 10,000 permutations and 15 degrees of freedom) are given for each analysis.
(61 KB DOC)

Table S2 Details of the 11 *GLI1* SNPs Genotyped in the Scottish Population, Including the Four tSNPs Used for Haplotype Analysis χ^2^ analysis for allelic frequency and homozygosity of each tSNP for UC and CD versus HCs is presented with corresponding *p*-values, OR, and 95% CIs. *, *n* = 370 for SNPs 1–4 and 7–9; U/K, unknown.
(90 KB DOC)

Text S1 Supplementary Methods (43 KB DOC)

## Abbreviations

CD: Crohn's disease
CI: confidence interval
DSS: dextran sodium sulphate
FPRP: false positive report probability
GLI1: glioma-associated oncogene homolog 1 (human)
Gli1: glioma-associated oncogene homolog 1 (mouse): HC, healthy controls
HH: hedgehog (human)
Hh: hedgehog (mouse)
HHIP: hedgehog-interacting protein
HWE: Hardy-Weinberg equilibrium
IBD: inflammatory bowel disease
IBDU: colonic IBD type unclassified
IHH: Indian Hedgehog
LD: linkage disequilibrium
OR: odds ratio
PTCH: patched
QPCR: quantitative reverse-transcription PCR
SHH: Sonic Hedgehog
SMO: smoothened
tSNP: tagging single nucleotide polymorphism
UC: ulcerative colitis
WT: wild type
