# Supplementary material for: Analysis of Germline GLI1 Variation Implicates Hedgehog Signalling in the Regulation of Intestinal Inflammatory Pathways
Source: PLoS Med. 2008 Dec 9;5(12):e239. doi: 10.1371/journal.pmed.0050239 (PMC2596854; doi:10.1371/journal.pmed.0050239)
Supplement: Supplementary file 1 [file pmed.0050239.sd001.doc]

**SUPPLEMENTAL METHODS**

**Sequencing and selection of tSNPs**

Sequencing of *GLI1* exons, intron / exon boundaries and 1kb of the promoter was performed in 32 patients with UC and CD to confirm the presence of dbSNPs (dbSNP build 124, 2005; pre-HapMap phase I data release) in the Scottish population prior to genotyping. Sequence was analysed using Sequencher 4.2 software (Gene Codes Corporation). Primer sets used to amplify *GLI1* are available on request.

Four multi-marker *GLI1* tSNPs (r^2^≥0.8) were identified (rs3817474, rs2228225, rs2228224, and rs2228226) based on SNP frequencies in the Scottish population to describe haplotypic variation of *GLI1*, detecting haplotypes of a frequency >1% (HaploView version 3.0).

**Note on population heterogeneity in Scotland**

We have previously reported low levels of genetic differentiation in terms of allele frequencies across mainland Scotland [2]. Since it has been shown that low levels of population stratification are likely only to result in weak associations [3] it is unlikely that the low levels present in the Scottish population could account for the associations between *GLI1* variation and disease risk which we report here.

**False positive report probability (FPRP)**

We estimated the posterior probability that the observed association (meta-analysis of *GLI1* SNP rs2228226) with disease risk represents a true positive by estimating 1 – FPRP using the method described by Wacholder [4] and the prior probabilities for a candidate gene study proposed by Newton-Cheh and Hirschhorn [5]. This approach is based on the fact that the probability of a false positive is dependent not only on the observed p value but also the statistical power of the study and the prior probability that the association is true. We made calculations using the spreadsheet written by El Ghormli and Wacholder and made freely available for non-commercial use [4].

**RT-PCR primers and probes**

The primers and probes for SHH were: forward cgcagctgctctaccaaata; reverse ggcttcagctggacttgac; probe tcctggacagcgaggccctg. The primers and probes for IHH, not present on the Agilent chip, were: forward cttcagcgatgtgctcattt; reverse ctgagtctcgatgacctgga; probe tactggaccgcgagccccac.

**Immunohistochemistry Methods for Human Tissue**

3μm sections from formalin-fixed, paraffin-embedded samples were dewaxed in xylene and rehydrated in graded alcohols. Antigen retrieval was performed in unmasking solution (H-3300, Vector Labs) at 1000W for 15 minutes. Endogenous peroxidase activity was blocked with 2% hydrogen peroxide (H_2_0_2_) for 15 minutes, endogenous avidin and biotin was blocked (Avidin/Biotin Blocking Kit, SP-2001, Vector Labs), and non-specific antibody binding was blocked with a serum-free protein block (X0909, DakoCytomation). All antibodies were diluted in DAKO diluent. Following H_2_0_2_ treatment, slides were loaded into Sequenza™ (Thermo Shandon) immuno-staining chambers. In between all steps slides were washed twice with PBS. Sections were incubated with primary antibodies overnight at 4°C. Primary antibodies used were anti-HH (N-19; 1:30, Santa Cruz Biotechnology). Biotinylated secondary antibodies (rabbit anti-goat, 1:400; DAKO) were applied at room temperature for 30 minutes, followed by avidin-biotin complex (Vector R.T.U. Vectastain Elite ABC Reagent, PK-7100). Substrate was visualised with diaminobenzidine (Liquid DAB+, DAKO), developed for 5 minutes. Negative controls were included for all antibodies and all samples with omission of primary antibody. SHH expression was blocked following pre-incubation (overnight at 4°C) with 5x blocking peptide (sc-1194P); no signal was detected with omission of the primary antibody (**Supplemental Figure 8**). However the close homology between SHH and IHH amino acid sequences means that we cannot exclude that N-19 is also detecting IHH protein.

**Phylogeny Analysis and Protein Alignment**

GLI1 protein sequences from several mammalian species (human, rhesus, mouse, rat, dog, and cow) were obtained from UCSC (genome.ucsc.edu) or Entrez Protein ([http://www.ncbi.nlm.nih.gov](http://www.ncbi.nlm.nih.gov/)). Sequences were loaded into MacVector (macvector.com) for alignment and analysis. Regional conservations scores were calculated individually by comparing the most different mammalian GLI1 proteins (human and mouse).

**Supplemental Reference List**

1.	Van Den Brink GR (2007) Hedgehog signaling in development and homeostasis of the gastrointestinal tract. Physiol Rev 87: 1343-1375.

2.	Vitart V, Carothers AD, Hayward C, Teague P, Hastie ND, Campbell H, Wright AF (2005) Increased level of linkage disequilibrium in rural compared with urban communities: a factor to consider in association-study design. Am J Hum Genet 76: 763-772.

3.	Wacholder S, Rothman N, Caporaso N (2000) Population stratification in epidemiologic studies of common genetic variants and cancer: quantification of bias. J Natl Cancer Inst 92: 1151-1158.

4.	Wacholder S, Chanock S, Garcia-Closas M, El Ghormli L, Rothman N (2004) Assessing the probability that a positive report is false: an approach for molecular epidemiology studies. J Natl Cancer Inst 96: 434-442.

5.	Newton-Cheh C, Hirschhorn JN (2005) Genetic association studies of complex traits: design and analysis issues. Mutat Res 573: 54-69.
