# Supplementary material for: Analysis of Germline GLI1 Variation Implicates Hedgehog Signalling in the Regulation of Intestinal Inflammatory Pathways
Source: PLoS Med. 2008 Dec 9;5(12):e239. doi: 10.1371/journal.pmed.0050239 (PMC2596854; doi:10.1371/journal.pmed.0050239)
Supplement: Supplementary file 2 [file pmed.0050239.st001.doc]

A.

| **HAPLOTYPE** | | | | | **IBD**  **n=809** | **UC**  **n=474** | **CD**  **n=335** | **HC**  **n=1374** | **IBD vs. HC**  p-value  OR (C.I.) | **UC vs. HC**  p-value  OR (C.I.) | **CD vs. HC**  p-value  OR (C.I.) |
| --- | --- | --- | --- | --- | --- | --- | --- | --- | --- | --- | --- |
|  | tSNP_1_ | tSNP_2_ | tSNP_3_ | tSNP_4_ |  |  |  |  |  |  |  |
| **A** | 1 | 1 | 1 | 1 | 59.2% | 59.6% | 58.7% | 60.9% | 0.26  0.93 (0.82-1.05) | 0.50  0.95 (0.81-1.10) | 0.30  0.91 (0.76-1.08) |
| **B** | 2 | 2 | 2 | 2 | 33.9% | 32.5% | 35.9% | 30.7% | **0.036**  1.16 (1.01-1.32) | 0.32  1.09 (0.93-1.28) | **0.012**  1.26 (1.06-1.51) |
| **C** | 2 | 2 | 2 | 1 | 4.9% | 4.8% | 5.1% | 6.9% | **0.014**  0.71 (0.54-0.93) | **0.031**  0.68 (0.49-0.96) | 0.13  0.74 (0.51-1.07) |
| **Log-likelihood p-value** | | | | | | | | | **< 0.0001** | **< 0.0001** | **0.03** |

B.

| **HAPLOTYPE** | | | | | **IBD**  **n=1748** | **UC**  **n=928** | **CD**  **n=737** | **HC**  **n=589** | **IBD vs. HC**  p-value  OR (C.I.) | **UC vs. HC**  p-value  OR (C.I.) | **CD vs. HC**  p-value  OR (C.I.) |
| --- | --- | --- | --- | --- | --- | --- | --- | --- | --- | --- | --- |
|  | tSNP_1_ | tSNP_2_ | tSNP_3_ | tSNP_4_ |  |  |  |  |  |  |  |
| **A** | 1 | 1 | 1 | 1 | 62.8% | 64.5% | 60.4% | 63.3% | 0.94  0.99 (0.87-1.13) | 0.48  1.06 (0.91-1.23) | 0.25  0.91 (0.78-1.06) |
| **B** | 2 | 2 | 2 | 2 | 29.7% | 30.8% | 28.2% | 27.7% | 0.14  1.12 (0.97-1.29) | 0.067  1.16 (0.99-1.36) | 0.61  1.05 (0.89-1.24) |
| **C** | 2 | 2 | 2 | 1 | 5.3% | 4.7% | 6.1% | 6.9% | **0.049**  0.76 (0.58-0.99) | **0.0093**  0.66 (0.49-0.90) | 0.44  0.88 (0.65-1.19) |
| **Log-likelihood p-value** | | | | | | | | | **0.009** | **< 0.0001** | **0.002** |

C.

| **HAPLOTYPE** | | | | | **IBD**  **n=493** | **UC**  **n=288** | **CD**  **n=205** | **HC**  **n=281** | **IBD vs. HC**  p-value  OR (C.I.) | **UC vs. HC**  p-value  OR (C.I.) | **CD vs. HC**  p-value  OR (C.I.) |
| --- | --- | --- | --- | --- | --- | --- | --- | --- | --- | --- | --- |
|  | tSNP_1_ | tSNP_2_ | tSNP_3_ | tSNP_4_ |  |  |  |  |  |  |  |
| **A** | 1 | 1 | 1 | 1 | 58.2% | 58.6% | 57.6% | 57.0% | 0.68  1.05 (0.85-1.30) | 0.62  1.07 (0.84-1.36) | 0.86  1.03 (0.79-1.35) |
| **B** | 2 | 2 | 2 | 2 | 35.1% | 34.4% | 36.1% | 34.2% | 0.81  1.03 (0.83-1.29) | 0.97  1.01 (0.78-1.29) | 0.55  1.10 (0.83-1.44) |
| **C** | 2 | 2 | 2 | 1 | 6.7% | 7.0% | 6.3% | 8.8% | 0.17  0.75 (0.50-1.10) | 0.32  0.78 (0.50-1.21) | 0.16  0.68 (0.41-1.12) |
| **Log-likelihood p-value** | | | | | | | | | 0.13 | 0.41 | 0.19 |
