# Supplementary material for: Analysis of Germline GLI1 Variation Implicates Hedgehog Signalling in the Regulation of Intestinal Inflammatory Pathways
Source: PLoS Med. 2008 Dec 9;5(12):e239. doi: 10.1371/journal.pmed.0050239 (PMC2596854; doi:10.1371/journal.pmed.0050239)
Supplement: Supplementary file 3 [file pmed.0050239.st002.doc]

| **SNP** | **dbSNP ID & Position** | **Location** | **Allele 1/2** |  | **UC**  **N=474** | **CD**  **N=335** | **HC**  **N=1374*** | **UC vs. HC**  P-value  OR (C.I.) | **CD vs. HC**  P-value  OR (C.I.) |
| --- | --- | --- | --- | --- | --- | --- | --- | --- | --- |
| 1 | rs2242578  56139420 | Promoter | C/G | *U/K*  CC  CG  GG  C  G | *48 (8.7%)*  221 (44.1%)  225 (44.9%)  55 (11.0%)  667 (66.6%)  335 (33.4%) | *44 (9.9%)*  164 (40.9%)  189 (47.1%)  48 (12.0%)  517 (64.5%)  285 (35.5%) | *30 (8.1%)*  151 (44.4%)  138 (40.6%)  51 (15.0%)  440 (64.7%)  240 (35.3%) | 22 vs 11  P=0.18  OR 0.74  (0.48-1.14)  2 vs 1  P=0.40  OR 0.92  (0.75-1.13) | 22 vs 11  P=0.57  OR 0.87  (0.55-1.36)  2 vs 1  P=0.96  OR 1.01 (0.82-1.25) |
| 2 | rs3825077  56142281 | Intron 1/2 | T/C | *U/K*  TT  TC  CC  T  C | *51 (9.3%)*  190 (38.2%)  249 (50.0%)  59 (11.8%)  629 (63.2%)  367 (36.8%) | *34 (7.6%)*  153 (37.2%)  201 (48.9%)  57 (13.9%)  507 (61.7%)  315 (38.3%) | *26 (7.0%)*  141 (41.0%)  152 (44.2%)  51 (14.8%)  434 (63.1%)  254 (36.9%) | 22 vs 11  P=0.51  OR 0.86  (0.56-1.32)  2 vs 1  P=0.94  OR 1.00  (0.82-1.22) | 22 vs 11  P=0.91  OR 1.03  (0.66-1.60)  2 vs 1  P=0.59  OR 1.06  (0.86-1.31) |
| 3 | rs4760148  56143043 | Intron 1/2 | G/C | *U/K*  GG  GC  CC  G  C | *65 (11.8%)*  209 (43.2%)  231 (47.7%)  44 (9.1%)  649 (67.0%)  319 (33.0%) | *40 (9.0%)*  175 (43.2%)  183 (45.2%)  47 (11.6%)  533 (65.8%)  277 (34.2%) | *20 (5.4%)*  171 (48.9%)  138 (39.4%)  41 (11.7%)  480 (68.6%)  220 (31.4%) | 22 vs 11  P=0.63  OR 0.88  (0.55-1.41)  2 vs 1  P=0.55  OR 1.07  (0.87-1.32) | 22 vs 11  P=0.72  OR 1.12  (0.70-1.79)  2 vs 1  P=0.27  OR 1.13  (0.91-1.41) |
| 4 | rs3782126  56143200 | Intron 1/2 | T/C | *U/K*  TT  TC  CC  T  C | *49 (8.9%)*  222 (44.4%)  233 (46.6%)  45 (9.0%)  677 (67.7%)  323 (32.3%) | *56 (12.6%)*  165 (42.4%)  185 (47.6%)  39 (10.0%)  515 (66.2%)  263 (33.8%) | *19 (5.1%)*  173 (49.3%)  137 (39.0%)  41 (11.7%)  483 (68.8%)  219 (31.2%) | 22 vs 11  P=0.55  OR 0.86  (0.54-1.37)  2 vs 1  P=0.67  OR 1.05  (0.86-1.29) | 22 vs 11  P=1.00  OR 0.99  (0.61-1.62)  2 vs 1  P=0.29  OR 1.13  (0.91-1.40) |
| **5**  ***tSNP_1_*** | **rs3817474**  **56145102** | **Intron 4/5** | **A/G** | ***U/K***  **AA**  **AG**  **GG**  **A**  **G** | ***26 (5.4%)***  **163 (35.9%)**  **232 (51.1%)**  **59 (13.0%)**  **558 (61.5%)**  **350 (38.5%)** | ***32 (9.6%)***  **105 (34.8%)**  **150 (49.7%)**  **47 (15.6%)**  **360 (59.6%)**  **244 (40.4%)** | ***95 (6.9%)***  **506 (39.6%)**  **577 (45.2%)**  **194 (15.2%)**  **1589 (62.2%)**  **965 (37.8%)** | **22 vs 11**  **P=0.81**  **OR 0.94**  **(0.67-1.33)**  **2 vs 1**  **P=0.71**  **OR 1.03**  **(0.88-1.21)** | **22 vs 11**  **P=0.49**  **OR 1.17**  **(0.80-1.71)**  **2 vs 1**  **P=0.25**  **OR 1.12**  **(0.93-1.14)** |
| **6**  ***tSNP_2_*** | **rs2228225**  **56145698** | **Exon 6** | **A/G** | ***U/K***  **AA**  **AG**  **GG**  **A**  **G** | ***37 (7.7%)***  **161 (36.2%)**  **220 (49.4%)**  **64 (14.4%)**  **542 (60.9%)**  **348 (39.1%)** | ***4 (1.2%)***  **118 (35.2%)**  **162 (48.4%)**  **55 (16.4%)**  **398 (59.4%)**  **272 (40.6%)** | ***91 (6.6%)***  **504 (39.3%)**  **577 (45.0%)**  **202 (15.7%)**  **1585 (61.8%)**  **981 (38.2%)** | **22 vs 11**  **P=0.96**  **OR 1.02**  **(0.74-1.42)**  **2 vs 1**  **P=0.67**  **OR 1.04**  **(0.89-1.21)** | **22 vs 11**  **P=0.46**  **OR 1.16**  **(0.81-1.67)**  **2 vs 1**  **P=0.28**  **OR 1.10**  **(0.93-1.31)** |
| 7 | rs2292657  56146199 | Intron 7/8 | A/G | *U/K*  AA  AG  GG  A  G | *39 (7.1%)*  186 (36.5%)  256 (50.2%)  68 (13.3%)  628 (61.6%)  392 (38.4%) | *38 (8.5%)*  149 (36.6%)  201 (49.4%)  57 (14.0%)  499 (61.3%)  315 (38.7%) | *26 (7.0%)*    146 (42.4%)  145 (42.2%)  53 (15.4%)  437 (63.5%)  251 (36.5%) | 22 vs 11  P=1.00  OR 1.01  (0.66-1.53)  2 vs 1  P=0.44  OR 1.09  (0.89-1.33) | 22 vs 11  P=0.82  OR 1.05  (0.68-1.63)  2 vs 1  P=0.39  OR 1.10  (0.89-1.36) |
| 8 | rs11830874  56147239 | Intron 8/9 | T/C | *U/K*  TT  TC  CC  T  C | *32 (5.8%)*  506 (97.9%)  11 (2.1%)  0 (0%)  1023 (98.9%)  11 (1.1%) | *29 (6.5%)*  404 (97.1%)  12 (2.9%)  0 (0%)  820 (98.6%)  12 (1.4%) | *16 (4.3%)*  340 (96.0%)  14 (4.0%)  0 (0%)  694 (98.0%)  14 (2.0%) | 22 vs 11  N/A  2 vs 1  P=0.075  OR 0.53  (0.24-1.18) | 22 vs 11  N/A  2 vs 1  P=0.61  OR 1.27  (0.64-2.52) |
| 9 | rs10783828  56147751 | Intron 9/10 | G/A | *U/K*  GG  GA  AA  G  A | *48 (8.7%)*  225 (44.9%)  229 (45.7%)  47 (9.4%)  679 (67.8%)  323 (32.2%) | *58 (13.0%)*  172 (44.4%)  169 (43.7%)  46 (11.9%)  513 (66.3%)  261 (33.7%) | *23 (6.2%)*  179 (51.6%)  128 (36.9%)  40 (11.5%)  486 (70.0%)  208 (30.0%) | 22 vs 11  P=0.81  OR 0.93  (0.59-1.49)  2 vs 1  P=0.35  OR 1.11  (0.90-1.37) | 22 vs 11  P=0.47  OR 1.20  (0.75-1.92)  2 vs 1  P=0.13  OR 1.19  (0.95-1.48) |
| **10**  ***tSNP_3_*** | **rs2228224**  **56151588** | **Exon 12** | **A/G** | ***U/K***  **AA**  **AG**  **GG**  **A**  **G** | ***14 (2.9%)***  **175 (37.2%)**  **230 (48.9%)**  **65 (13.8%)**  **580 (61.7%)**  **360 (38.3%)** | ***14 (4.1%)***  **109 (33.6%)**  **158 (48.8%)**  **57 (17.6%)**  **376 (58.0%)**  **272 (42.0%)** | ***101 (7.4%)***  **506 (39.7%)**  **560 (44.0%)**  **207 (16.3%)**  **1572 (61.7%)**  **974 (38.3%)** | **22 vs 11**  **P=0.62**  **OR 0.91**  **(0.65-1.26)**  **2 vs 1**  **P=0.98**  **OR 1.00**  **(0.86-1.17)** | **22 vs 11**  **P=0.21**  **OR 1.28**  **(0.89-1.83)**  **2 vs 1**  **P=0.091**  **OR 1.17**  **(0.98-1.39)** |
| **11**  ***tSNP_4_*** | **rs2228226**  **56152088** | **Exon 12** | **C/G** | ***U/K***  **CC**  **CG**  **GG**  **C**  **G** | ***9 (1.9%)***  **205 (43.2%)**  **217 (45.7%)**  **53 (11.2%)**  **627 (66.0%)**  **323 (34.0%)** | ***10 (2.9%)***  **137 (41.5%)**  **148 (44.8%)**  **45 (13.6%)**  **422 (63.9%)**  **238 (36.1%)** | ***110 (8.0%)***  **610 (48.3%)**  **542 (42.9%)**  **112 (8.9%)**  **1762 (69.7%)**  **766 (30.3%)** | **22 vs 11**  **P=0.079**  **OR 1.41**  **(0.98-2.03)**  **2 vs 1**  **P=0.040**  **OR 1.19**  **(1.01-1.39)** | **22 vs 11**  **P=0.0048**  **OR 1.79**  **(1.21-2.65)**  **2 vs 1**  **P=0.0053**  **OR 1.30**  **(1.08-1.55)** |
